# An exploratory fMRI study on the association of parental involvement in childcare with brain responses to infant vocalisations and dyadic interaction quality in same-sex mother families

**DOI:** 10.1186/s40359-025-03439-9

**Published:** 2025-10-07

**Authors:** Paola Rigo, Michele Giannotti, Bianca Filippi, Alessandra Simonelli, Micol Gemignani, Silvia Perzolli, Paola Venuti, Simona de Falco

**Affiliations:** 1 Department of Developmental Psychology and Socialisation, University of Padua, Via Venezia 8, Padua, Padova, 35131 PD Italy; 2 Department of Psychology and Cognitive Science, University of Trento, Corso Bettini 84, Rovereto, 38068 TN Italy; 3 Faculty of Psychology, eCampus University, Novedrate, CO Italy

**Keywords:** Same-sex families, Maternal brain, Infant vocalisation, Caregiver involvement, Parent-child interaction

## Abstract

It is of the utmost importance that children receive an appropriate adult response in order to ensure optimal child development. Prior research on parental brain networks has demonstrated that maternal brain activation in response to infant vocalisations exhibits a positive correlation with observed maternal sensitivity. However, the neurobiological foundations of responsiveness to child cues in mothers of same-sex families remain unexplored. Such an investigation could contribute to the expansion of the existing knowledge base regarding parental brain responsiveness and its relationship with factors associated with sensitive parenting, such as caregiving involvement. Twenty-one same-sex mothers were subjected to an fMRI listening task comprising positive and negative infant vocalisations. The degree of maternal involvement in the care of one’s own child and the quality of parent-child dyadic interactions (measured by the Emotional Availability (EA) Scales) were assessed. The results indicated a positive correlation between the degree of maternal involvement in childcare and maternal sensitivity and non-intrusiveness. Furthermore, mothers who demonstrated high levels of EA structuring and non-hostility during dyadic interactions exhibited increased activation in the right midbrain, temporal pole, extended to the insular cortex, in response to infant negative vocalisations. Finally, a consistent positive correlation was observed between the activation of the putamen in response to positive infant vocalisations and both the degree of maternal involvement in childcare and most of the maternal EA scales. Overall, preliminary findings in same-sex mothers suggest that optimal mother-child emotional connection is associated with time spent with and for the child in daily activities and with maternal brain responses to infant sounds in regions underlying emotion regulation, motivation and salience processing.

**Supplementary Information:**

The online version contains supplementary material available at 10.1186/s40359-025-03439-9.

## Introduction

An ever-growing number of neuroscience studies have highlighted how parental behaviour, observable in adult-child interactions [1, 2], is partly determined by specific neural activity in response to infant sensory signals. Newborns, and children in general, can elicit the attention, closeness, and care of adults through emotional vocalisations [3]. Vocalisations of discomfort or joy inform about the child’s state and solicit care actions oriented toward the expressed needs [4, 5, 6, 7]. Research on the parental brain has uncovered cognitive and affective neurobiological mechanisms associated with the ability of listeners to encode and process the child faces or cries in childless adults [8, 9, 10, 11] and parents [10, 12, 13, 14, 15, 16], understand a child’s underlying needs, and, therefore, respond differently to these needs [17, 18, 19, 20, 21, 22, 23]. One of the most profound contributions of such research has highlighted that human parenting, encompassing care and protection that adults provide to children, has no less of a biological basis than other crucial skills oriented towards survival, which is the primary objective of all species [3, 24, 25, 26, 27, 28, 29, 30]. 

Crying is one of the fundamental and primary skills that allows infants to communicate their needs and it is, therefore, one of the first signals that parents are led to respond [5, 31, 32, 33]. According to the biological relevance of infant crying, several fMRI studies have focused on maternal brain reactions to infant crying from familiar children (i.e [14, 17, 25, 34, 35, 36, 37, 38]. Infant cry perception may recruit several brain networks in mothers to support acoustic analysis (auditory pathways), attentional shifting (thalamo-cingulate pathway), salience-emotional appraisal and mentalising (insula, dorsomedial prefrontal cortex, inferior frontal gyrus, pars triangularis and orbitofrontal gyrus), as well as reward-motivation processing (basal forebrain and mesolimbic and mesocortical dopaminergic pathways). It may also engage networks involved in the initiation and planning of motivated behaviours (medial and lateral motor networks with basal ganglia, medial superior frontal gyrus) (for a review see i.e [5, 20, 27, 39, 40, 41, 42, 43]). A recent systematic meta-analysis of neuroimaging data examined the cerebral circuits activated by familiar and unfamiliar infant cry perception in human adults, and then the moderating effect of gender and parental status (parent vs. nonparent) [44]. The overall results of the study confirmed previous hypothesised models of the neural underpinnings of parental response to infant crying in human adults, both parents and nonparents, consistent with an evolutionary perspective [5] on the biological salience of infant crying to guide adult caregiving behaviour. However, Witteman and colleagues [44] also argued that biological female adults and parents showed a different activation gradient of integrated networks of cortico-limbic and sensorimotor structures compared to males and nonparents. Speculatively, the authors suggested that these findings may potentially support more efficient processing of - and thus more motivated emotional responses to - such salient infant vocalisations in females and parents. Indeed, Kim and colleagues found cross-cultural evidence linking maternal brain response to infant crying, underlying motor and auditory processing, with cortisol levels, regardless of familiarity, and maternal intrusiveness during dyadic mother-child interactions [17]. The evidence discussed so far suggests a link in mothers between brain activation when listening to infant crying, independent of familiarity, and the quality of their maternal behaviour toward their child. Indeed, studies on parental brain networks have shown that brain activations in response to an infant’s cry positively correlate with observed maternal sensitivity [45, 46, 47]. Specifically, the activation of the right superior frontal gyrus, the amygdala, the right frontopolar and the inferior frontal regions have been associated with optimal maternal responses to infant distress signals [14, 48]. In addition, parental neural activation in response to their own infant’s cry sound has been related to infant attachment security and behaviours [36, 49].

Although there has been no systematic study on the brain response to positive infant vocalisations other than in comparison to infant crying, research has shown that infant laughter elicits a greater response in brain regions involved in emotional salience appraisal, mentalising, and initiation and planning of motivated behaviour. These regions include the right amygdala and middle cingulate cortex, bilateral insula, left ventral prefrontal cortex and temporoparietal junction [50, 51, 52, 53]. Shifting the focus to other sensory modalities, fMRI studies investigating positively valenced infant stimuli have shown that infants’ happy faces, in contrast to neutral or sad expressions, activate brain regions involved in motivation, salience, emotional regulation, and mentalisation [54]. These brain regions play a crucial role in initiating and facilitating parental caregiving responses to infants, fostering attentive and nurturing behaviour [48, 55].

Overall, neuroimaging studies of parental response to infant vocalisations have focused predominantly on mothers from traditional different-sex families, historically considered the child’s primary caregiver. However, the concept of family has broadened to encompass various structures (56–60), including same-sex families. In these cases, both adults are responsible for caring for and protecting the child, although they may not be biologically related to the child. Interestingly, same-sex parents tend to share caregiving responsibilities and duties more equally compared to different-sex parents families [61], and their inclusion in research could also help to disentangle the role of caregiving involvement in modulating neurobiological responses to child stimuli [39, 62, 63].

Following this perspective, the first neurobiological findings on families with same-sex parents [62] demonstrated that, when exposed to visual stimuli of their own adult-child interactions, gay fathers, as primary caregivers in same-sex families, showed a brain activation pattern that combines the typical brain activation patterns of mothers and fathers, in different-sex families. Other research has indeed shown that primary-caregiver gay fathers show substantial involvement in physical play, typically observed in father-child interactions [64], as well as strong emotional support for children historically been linked to maternal parenting [65]. For example, primary-caregiving fathers show greater dyadic synchrony than secondary-caregiver fathers [62]. Research has suggested that the quality of parenting depends more on psychological dimensions such as warmth and sensitivity than on family structure, and that parental role may be more determined by involvement in childcare [65, 66, 67, 68, 69]. Although initial knowledge exists on the impact of caregiver involvement in childcare on brain responsivity and parent sensitivity in gay fathers, much less is known about same-sex families of mothers [70]. Indeed, to our knowledge, the neurobiological bases of mothers’ responsiveness to children’s cues in same-sex families have never been studied. This would extend the generalisability of the empirical results on the parental brain model and contribute to understanding the factors associated with sensitive parenting, such as caregiving involvement, in all family forms.

In this preliminary study, we therefore investigated the parental brain response to emotional infant vocalisations in a group of same-sex mothers and its relationship to levels of maternal sensitivity during mother-child interactions and caregiving involvement in childcare. Maternal sensitivity and caregiving involvement in childcare are two psychological constructs that are considered to be of primary importance in characterising the quality of parenting [45, 62, 69, 71]. To ensure the inclusion of a wide range of maternal experiences, the sample of mothers was recruited regardless of whether they were biological or non-biological caregivers.

We measured parental brain response during a listening task to positive and negative emotional infant vocalisations. We used a set of standardised infant sounds which, due to the intrinsic biological salience of infant vocalisations, can elicit maternal brain activations consistent with the parental brain model [17, 25, 44]. We expected that the brain activation underlying the emotional and motivational processing of salient infant cues would be associated with maternal sensitivity during dyadic interactions. This is because sensitivity is assumed to rely on the ability to recognise and empathise with an infant’s emotional signals. In line with previous findings that highlight brain malleability in relation to caregiving experience [62, 72, 73, 74, 75], we also expected to find a significant association between the level of involvement in childcare and activation in the brain regions underlying emotion-motivation processing networks [62]. We also expected that caregiving involvement would be positively related to the quality of mother-child interactions. Indeed, previous literature [62] has reported that mothers and primary caregiving fathers exhibit an interactive style characterised by greater parental behaviour in accordance with, and attuned to, the infant’s social signals, as well as greater parent-infant synchrony during dyadic interactions.

## Materials and methods

### Participants

A total of thirty-two same-sex mothers (age *M* = 43.38, *SD* = 6.39) were recruited. Exclusion criteria were having history of psychiatric disorders, and being non-MR compatible. Among these parents, 11 were excluded from the fMRI session for MR-compatibility issues (i.e., tattoos, biomedical implants, and devices inside the body). The final sample was composed of 21 mothers (age *M* = 42.62, *SD* = 6.00) and 13 children (age *M* = 4.69, *SD* = 2.39; 7 females). The duration of the relationship with the partner was as follows (years range (percentage)): 6–10 (23.80%), 11–15 (38.10%), and 15 ≥ (38.10%). Sixteen of the mothers were married to each other, comprising eight couples. Mothers had high-level education on average (42.86*%* high-school diploma; 52.38*%* degree or higher). In the sample, 69.23*%* of mothers were first-time parents (Table 1). All included mothers were Italian. The 11 mothers excluded from the fMRI session were comparable with the 21 included mothers with regard to all the variables of interest (demographics, caregiving involvement, and quality of mother–child interaction). Except for cultural background, no statistical differences emerged. Two of the excluded mothers had dual nationality (Italian-American and Polish-British). The study protocol (No. 2020‑034) received approval from the Ethics Committee of the University of Trento and was conducted in accordance with the Declaration of Helsinki.

### Procedures

Prior to participating, all participants provided their informed consent, as well as that of their minor children. Additionally, we verbally obtained the assent of the children involved in the interactive session. Before each step of the study, each task was briefly described to participants. First, mothers were asked to complete online self-report questionnaires and were instructed to complete the questionnaires separately. Next, a trained researcher visited “virtually” (Zoom platform) families’ homes for the mother-child play interaction session; two home visits were scheduled in families where both mothers participated in the present study. The visits were scheduled based on the mothers’ availability. In advance, researchers sent age-appropriate puzzles to the families. A twelve-minute play interaction between mother and child with the puzzle was recorded. Finally, mothers underwent a neuroimaging session at the University of Trento. The fMRI scans were conducted on the same day for each mother (when both mothers in a family participated). The interval between behavioural and fMRI data collection sessions for each family was approximately two to three months. The overall data collection period for the study was from March 2022 to October 2023.

### Caregiving involvement (in childcare)

To assess caregiving involvement in childcare, mothers completed a 10-item questionnaire adapted from the structured interview of Parents’ Primary Caregiving Role [76]. The 10 items covered multiple activities: (1) Playing or talking or reading with child; (2) Getting child ready for bed, school or other activities; (3) Reviewing/helping with child’s school work; (4) Teaching a child skills and things about the world (outside of school); (5) Getting up during the night with a child; (6) Staying home with a sick child; (7) Making childcare arrangements; (8) Chauffeuring children; (9) Out-of-home child-related activities or functions (with or without children; e.g., doctor visits, PTA); (10) Coordinating and planning child or family activities (e.g., planning pick-ups, drop-offs, scheduling, making reservations). For each activity, mothers were asked to select the level of responsibility on a 5-point Likert scale (1 = none or very little responsibility, 2 = some responsibility, 3 = about half of the responsibility, 4 = much responsibility, 5 = almost complete or complete responsibility). Moreover, for each statement, mothers were asked to give three answers, referring to (i) their own level of responsibility; (ii) their partner’s level of responsibility; (iii) other people’s level of responsibility (e.g., grandparents, babysitters). Items have been translated into Italian and back-translated by a native English speaker. In cases where parents had multiple children, they were asked to refer to the child involved in the research. In the present study, only the scale assessing their own level of responsibility for childcare (sum of the 10 items “i”) was used. Cronbach’s alpha for this scale was satisfactory (α = 0.86).

### Emotional availability scales (EA scales)

Mother-child interaction was video-recorded and coded according to the fourth version of the Emotional Availability Scales [46], which allows the assessment of six dimensions that take into account both the contribution of the adult (i.e., parenting behaviours) and the child. The adult dimensions are sensitivity, structuring, non-intrusiveness, and non-hostility. The child dimensions are responsiveness (to the adult) and involvement, the latter reflecting the child’s active attempt to engage the adult. Specifically, the sensitivity scale measures the caregiver’s ability to be warm and emotionally connected with the child; the structuring scale assesses the caregiver’s ability to scaffold the child’s activities and set appropriate limits; the non-intrusiveness scale evaluates the caregiver’s ability to be available to the child without being intrusive, while the non-hostility scale measures the degree of overt and covert hostility toward the child. Child responsiveness and involvement scales refer, respectively, to the child’s ability to respond to the adult and be emotionally regulated and to the child’s own initiative to engage the adult. Each scale can be rated globally on a direct score ranging from 1 to 7. More specifically, scores between 5.5 and 7 are considered adequate and index a healthy relationship; scores around 4 indicate inconsistency (i.e., behaviours that are appropriate in some ways but that are not entirely healthy); scores of 3 or below refer to less optimal interactions where problematic behaviours can be observed. The system is applicable across a wide developmental age span (e.g., infancy into early adolescence), allowing for overall scores that can be compared with each other [77]. Two independent trained raters using the authorised training system coded the videotaped interactions. The inter-rater reliability was calculated using the Intraclass Correlation Coefficient (ICC) on a set of 6 videos for each EA dimension (sensitivity ICC = 0.74; [95% CI: 0.042 < ICC < 0.957]; structuring ICC = 0.69 [95% CI: -0.052 < ICC < 0.948]; non-intrusiveness ICC = 0.69 [95% CI: -0.048 < ICC < 0.949]; non-hostility ICC = 0.77 [95% CI: 0.114 < ICC < 0.963]; responsiveness ICC = 0.79 [95% CI: 0.177 < ICC < 0.967]; involvement ICC = 0.84 [95% CI: 0.304 < ICC < 0.975]). The first trained rater coded the entire dataset, while the second trained rater coded a subset of 6 (29%) randomly selected videos (more than the recommended 20% of the total sample was coded by two coders). Random selection ensured that potential bias due to the first rater coding interactions involving the same child with both mothers was minimised. When a difference of more than one point was seen in the coding scores of the coders (for example, a rating of 3 was given by one coder and a rating of 4.5 was given by the other), the consensus would have been reached through discussion. They met five times. The scores of the first rater were used for all subsequent analyses.

### Acoustic stimuli

Acoustic stimuli consisted of 10 crying (CS) and 10 laughing (LS) sounds from infants aged 1–2 years, and 10 control noise sounds (CNS). The infant vocalisations were downloaded from public online databases (https://audiojungle.net/, www.sounddogs.com; www.soundbible.com; www.audio4fun.com; www.freesound.org). CNSs were derived from ICSs. Following the generation of 10 white noise sounds, the noise signal’s shape and form were modulated using CSs as a reference point, with the objective of preserving the morphological features of the temporal pattern expressed by CSs. All stimuli were equated for volume. All acoustic files were processed using Audacity 2.4.1 (www.audacity.sourceforge.net) and Adobe Audition CC 2015 (Adobe Systems Incorporated, https://creative.adobe.com/products/audition) software. To validate the sounds, 70 adults (age *M* = 43.03, *SD* = 15.74; 42 female) rated the emotional valence of the sounds on a 7-point Likert scale ranging from 1 (extremely negative) to 7 (extremely positive). Results from a repeated-measures ANOVA showed a significant difference in the perceived emotional valence of CS, LS and CNS (*M*_CS_ = 2.63 ± 1.14, *M*_LS_ = 5.95 ± 0.84, *M*_NCS_ = 3.27 ± 0.88; *F*_(2, 138)_ = 282, *p* < .001, *η*^*2*^ = 0.69), with CS rated as significantly more negative than LS (*M*_difference_ = -3.32, *p*_*Tukey*_ < 0.001) as well as than CNS (*M*_difference_ = -0.64, *p*_*Tukey*_ < 0.001), and LS rated as significantly more positive than CNS (*M*_difference_ = 2.68, *p*_*Tukey*_ < 0.001).

### fMRI protocol

**Fig. 1:**
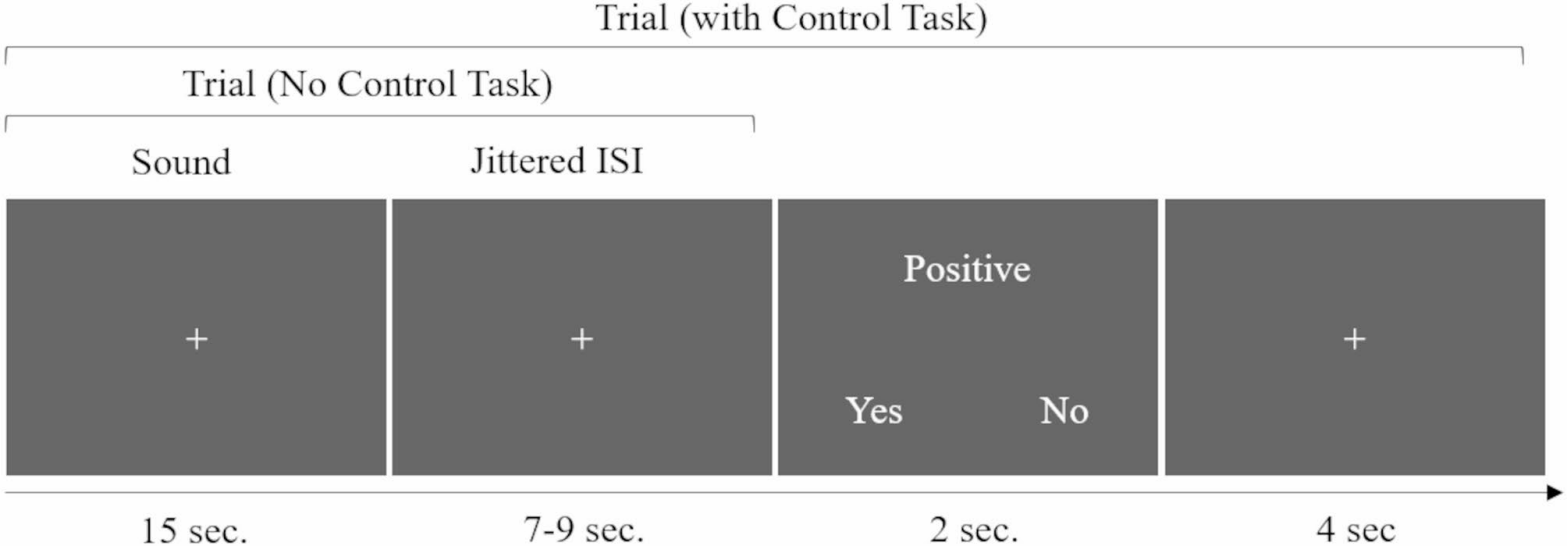
Experimental set-up of the listening task. At the end of each sound, the interstimulus interval was jittered. To ensure the participants’ attention, after 6 trials, they were asked to rate the valence of the sounds to ensure their attention to the task

During functional scanning, mothers underwent a passive listening task and were instructed to attend to all stimuli, namely infant and noise sounds (Fig. 1). Mothers listened to sounds presented binaurally using a Siemens foam ear plug connected to the pneumatic audio system of the MR (for stimuli presentation and scanner noise attenuation). All sounds lasted 15 sec, with an inter-stimulus interval of 7–9 sec, during which no auditory stimuli were presented (rest). At the beginning of the listening task, a fixation point was back-projected onto a 32” Nordic NeuroLab liquid crystal display monitor and continuously presented until the end of the task. Each sound was presented only once, and the presentation order was randomised for each participant. To ensure full attention to all sounds, after 6 trials, mothers were asked to evaluate the emotional valence of the sounds; mothers were instructed to press either a left or right button (namely yes and no) after being presented with one of the following questions: ‘positive?’ or ‘negative?’ or ‘neutral?’. All mothers attended a training session to familiarise them with the experimental task before being scanned. Each sound block (sound and rest) lasted between 22 and 24 s. and was presented in a random order. The duration of the task was about 11 min.

### fMRI data acquisition

All MR images were recorded through a whole body Siemens Prisma 3T magnet, 80mT/m gradients with a slew rate of 200 T/m/s, equipped with a 20 channels RF coil and a 64 channels RF coil. First, functional T2*-weighted images were obtained with a standard echo planar imaging (EPI) technique slice thickness = 3 mm, field of view (FOV) = 192 mm, flip angle = 75°, TR (repetition time) = 2000 ms, TE (echo time) = 28 ms, matrix 64 × 64, voxel size 3 × 3 × 3 mm, 368 volumes). The functional session lasted about 11 min. Second, we acquired high-resolution T1-weighted MPRAGE structural images (176 axial slices, slice thickness = 1 mm, FOV = 256 mm, TI (inversion time) = 1100 ms, flip angle = 7°, TR = 2530 ms, TE = 1.69 ms).

### fMRI data analysis

All MRI data analyses were performed with Statistical Parametric Mapping Software SPM12 (Functional Imaging Laboratory, UCL Queen Square Institute of Neurology, London, UK) running on a Windows operating system. To avoid the magnetic saturation effect, the task scan session was preceded by four dummy volumes. To preprocess the BOLD signal, we followed a default pipeline. T2-weighted functional images were realigned to the first functional image as a reference and then corrected for head movements. The realigned functional images’ mean and T1-weighted MPRAGE images were co-registered. The functional realigned and coregistered images in native space were normalised to the stereotaxic standard space Montreal Neurological Institute (MNI). The normalised images were spatially smoothed, using an 6-mm full-width half-maximum Gaussian kernel, and then a high-pass filter with 128 s cut-off was applied to remove low-frequency drifts.

For each participant, analytic design matrices were generated to model the onsets and duration of each sound as epochs convolved with a hemodynamic response function.

A fixed-effects general linear model (GLM) was implemented at the first-level statistical analysis to calculate the whole brain statistical map for each participant. To estimate the hemodynamic response amplitudes, three standard regressors, one predictor for each condition (CS, LS, and NCS), with time derivatives, were built by convolving a boxcar function with the standard canonical hemodynamic response function. To account for the head motion effect and autocorrelations in the timeseries, we included the six head motion parameters and employed an autoregressive AR [1] model.

A random-fixed second-level analysis was implemented on the group level to generalise results to the population. From the first-level analysis, we imported one sample t-test for the following contrast images: CS > NCS, CS < NCS, LS > NCS, LS < NCS, CS > LS and CS < LS. We used t-tests to estimate the significant difference from zero between the experimental conditions of interest [78]. Age of mother and child were included as covariates. The second-level SPM map was built at the threshold of voxel-level *p* < .0005 uncorrected. Cluster-level significant results were set at a threshold of *p*_(FWE)_ < 0.05 corrected for multiple comparisons (family-wise error FWE). Next, beta values of significant clusters resulting from the cluster-level analysis were extracted to statistically test the relationship between participants’ significant brain activity and psychological measures. WFU PickAtlas [79] and Xjview (https://www.alivelearn.net/xjview), SPM 12 extension toolboxes, were used for result mapping and visualisations. We used the NeuroPower software (http://neuropowertools.org/) to perform a post hoc power analysis. With the parameters of the present study, the power was found to be less than 10%, which confirms the preliminary nature of the study.

## Results

### Descriptives and preliminary analysis

Data were analysed using the statistical software Jamovi Version 2.3.28.0. We computed descriptive statistics to explore the characteristics of participants (Table 1), Caregiving Involvement and EAS (Table 2). We analytically tested the distribution of the variables of interest (e.g., Caregiving Involvement and EAS) using the Shapiro-Wilk test and, with the exception of EA sensitivity (*W* = 0.95, *p* = .344) and structuring (*W* = 0.93, *p* = .123), the variables did not follow a normal distribution. Next, preliminary correlation analyses were carried out to test potential confounds of demographic variables (mothers: age and number of children, education, relationship length with the partner; children: age and sex) with measures of Caregiving Involvement and EAS scores (Supplementary Information: Tables S1 and S2). Education level was negatively associated with Caregiving Involvement (*rho* [19] = -0.44, *p* < .048).

**Table 1:** Sociodemographic characteristics of participants

Variable		*N*	Mean or Percentage (range)	SD
Mothers’ age (years)		21	42.62 (28–55)	6.00
Child’s age (years)		13	4.69 (3–11)	2.39
Parity (1 child)		9	69.23%	–
Nationality (Italian)		21	100%	–
Education				–
	Middle school	1	4.76%	–
	High school	9	42.86%	–
	Bachelor degree	2	9.52%	–
	Master degree	5	23.81%	–
	PhD/post-graduate	4	19.05%	–
	Employment status (employed)	21	100%	–
Relationship duration (partner)				–
	6–10 years	5	23.80%	–
	11–15 years	8	38.10%	–
	15 + years	8	38.10%	–

**Table 2:** Descriptives of caregiving involvement and EA scales

Variable		Mean	SD
Caregiving Involvement		33.10	6.62
EAS			
	Sensitivity	5.63	0.86
	Structuring	5.63	1.09
	Non-intrusiveness	6.25	0.94
	Non-hostility	6.68	0.57
	Child Responsiveness	5.53	0.91
	Child Involvement	5.45	1.11

### Caregiving involvement in the childcare and EAS correlations

Given previous findings in same-sex families [62], we expected a positive association between caregiving involvement and high levels of EA. In light of the previous point, in order to assess the positive linear relation between the responsibility degree in child care (Caregiving Involvement) and the quality of dyadic EA during mother-child interaction (EAS), we computed one-tail nonparametric correlations (Spearman’s *rho*) to increase the power to detect the effect given the small sample size. Analysis revealed that the more involved mothers showed high sensitivity and non-intrusiveness, and children showed high responsive scores with more involved mothers (Table 3).

**Table 3:** Correlation between caregiving involvement in the childcare and EA scales

	1		2		3		4		5		6		7
**1** Caregiving Involvement	—												
**2** Sensitivity	0.40	*	—										
**3** Structuring	0.32		0.79	***	—								
**4** Non-intrusiveness	0.67	***	0.80	***	0.69	***	—						
**5** Non-hostility	0.20		0.70	***	0.53	**	0.48	*	—				
**6** (Child) Responsivity	0.39	*	0.81	***	0.74	***	0.70	***	0.46	*	—		
**7** (Child) Responsivity	0.35		0.77	***	0.80	***	0.64	**	0.47	*	0.91	***	—

Note. Hₐ is positive correlation

Note. * *p* < .05, ** *p* < .01, *** *p* < .001, one-tailed (uncorrected for multiple comparisons), the correlation between Non-intrusiveness and Caregiving Involvement survived to the Bonferroni-adjusted *p*-value threshold of *P* < .008

### fMRI results

**Fig. 2:**
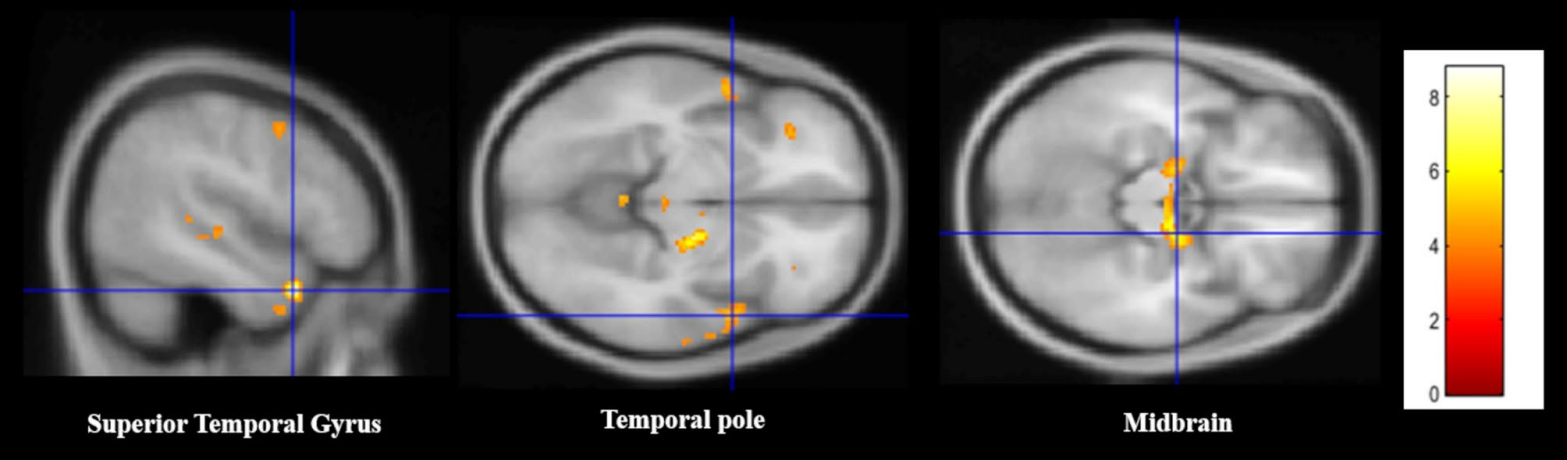
Infant cry sound (CS) vs. control noise sound (NCS) contrast: Activated cluster peaks with Brodmann areas (BA), side (R = right; L = left), MNI coordinates (x, y, z), cluster sizes and t–values were reported

In *CS > NCS contrast*, the analysis revealed activated cluster peaks in the right superior temporal gyrus STG (pole; BA 28), midbrain (extended to bilateral hippocampus and amygdala), and the pole of STG (extended to insula). In the *CS < NCS contrast*, no significant deactivated clusters were found. (Fig. 2; for peaks of brain activity and cluster size, see Table 4).

**Fig. 3:**
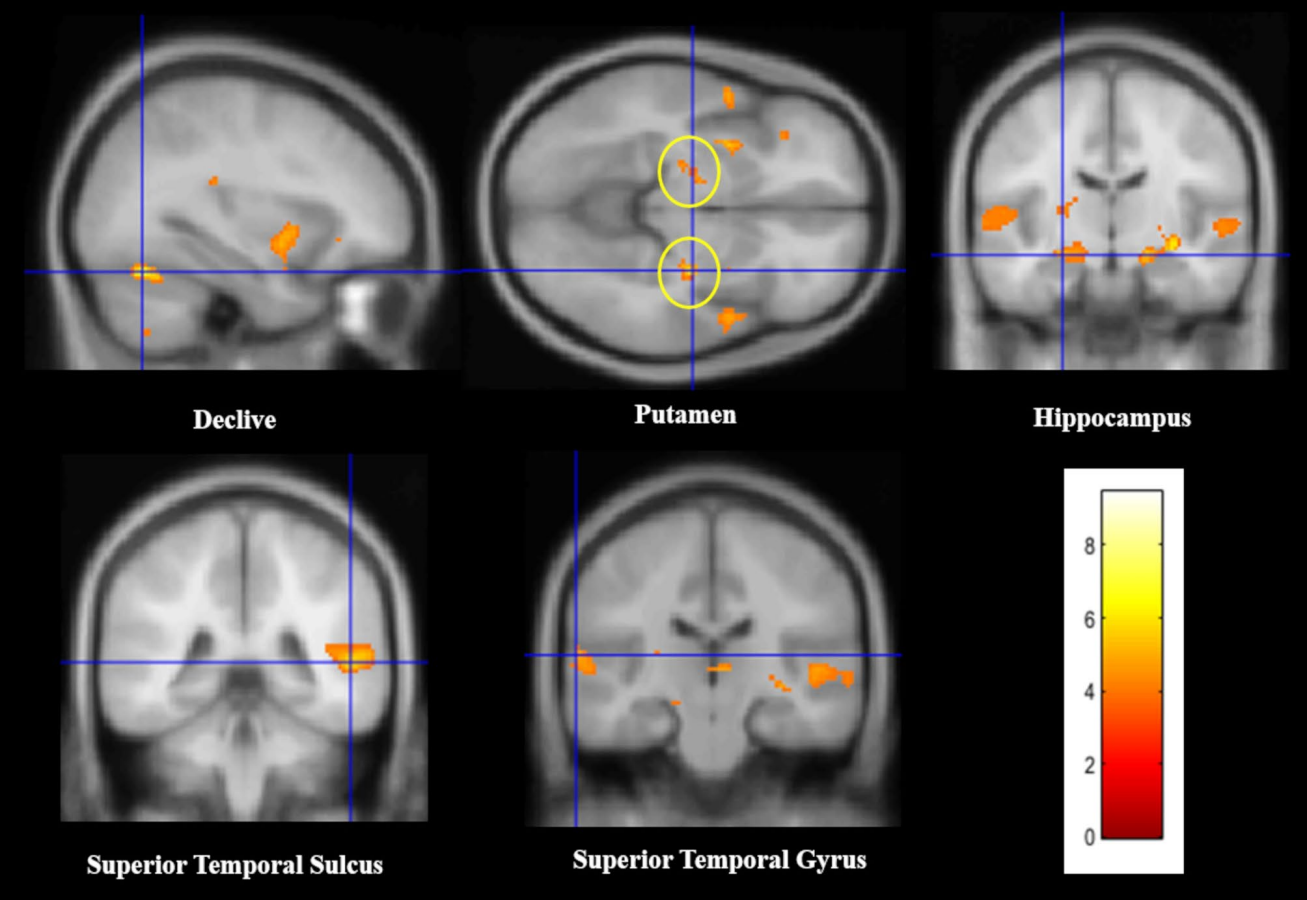
Infant laugh sound (LS) vs. control noise sound (NCS) contrast: Activated cluster peaks with Brodmann areas (BA), side (R = right; L = left), MNI coordinates (x, y, z), cluster sizes and t–values were reported

In *LS > NCS contrast*, the analysis showed activated cluster peaks in the left putamen, declive (extended to the fusiform gyrus, BA 37), hippocampus (extended to parahippocampus and substantia nigra SN, BA 34), and STG (BA 22). In the right hemisphere, activated cluster peaks were found in the putamen (extended to the hippocampus and amygdala, BA 38) and sulcus of the STG (extended to the medial temporal gyrus MTG). In the *LS < NCS contrast*, no significant deactivation has been observed (Fig. 3; for peaks of brain activity and cluster size, see Table 5).

**Fig. 4:**
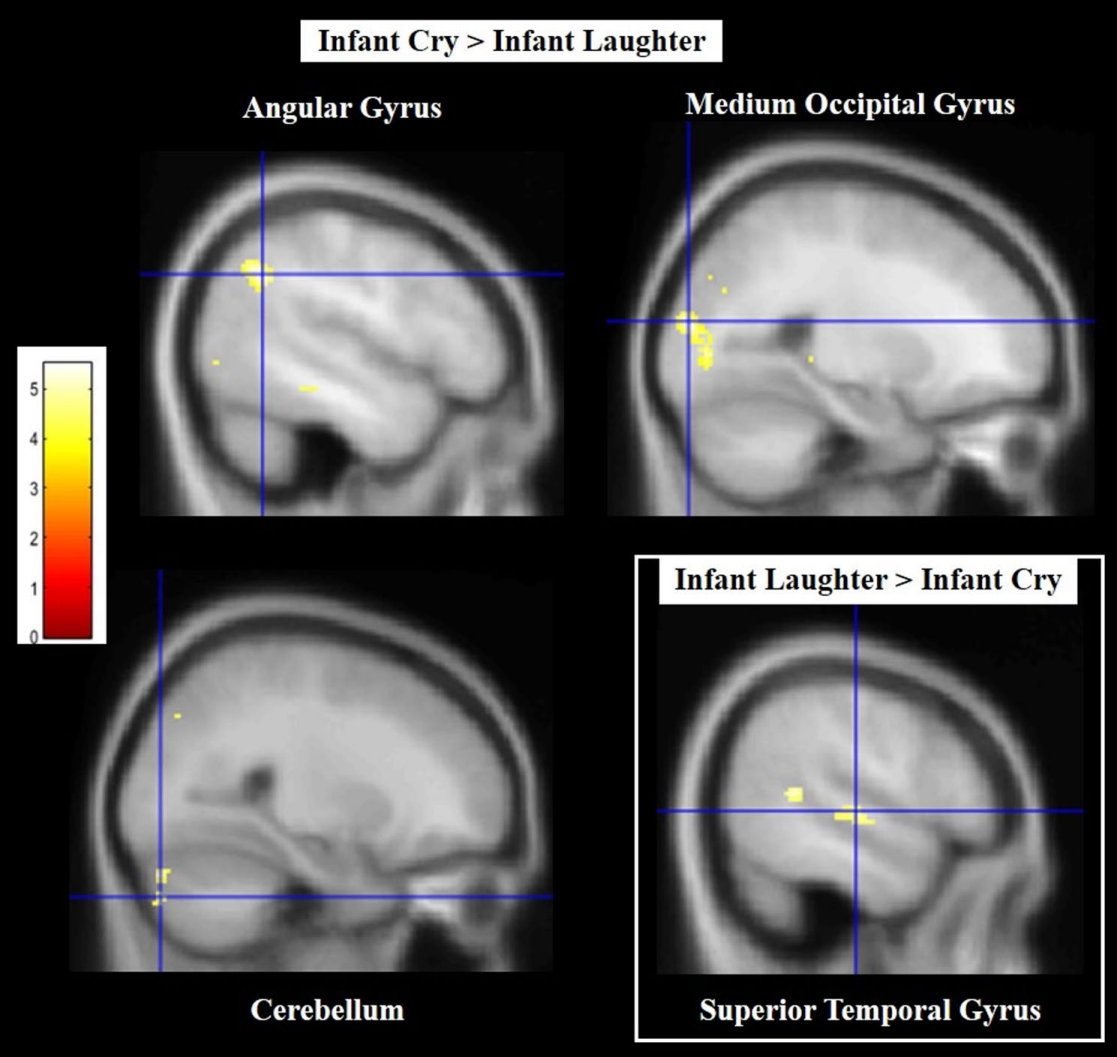
Infant cry sound (CS) vs. infant laugh sound (LS) contrast: Activated cluster peaks with Brodmann areas (BA), side (R = right; L = left), MNI coordinates (x, y, z), cluster sizes and t–values were reported

In *CS > LS contrast*, the analysis showed activated cluster peaks in the left angular gyrus (BA 40; extended to the supramarginal gyrus) and MOG (BA 18/19; extended to cuneus), and the right cerebellum (Crus 1). In *CS < LS contrast*, a significant deactivated cluster peak was found in the right STG (BA 22) (Fig. 4; for peaks of brain activity and cluster size, see Table 6).

**Table 4:** Maternal brain activation in response to infant crying sounds (CS) contrasted to control noise sounds (NCS). Cluster peaks of cerebral activity with brodmann areas (BA), coordinates in MNI space (*X*,* Y*,* Z*), cluster sizes, and *t*-values at *p*(FWE) < 0.05

Regions			MNI coordinates			Cluster size	*t*-value	
			(peak within a cluster)					
	BA	Side	X	Y	Z			
STG (temporal pole)	28/34	R	48	14	-30	144	8.79	
Midbrain (bilateral hippocampus, amygdala)	28/34	R	18	-10	-12	374	8.57	
pole STG (insula)	21/22/38	R	54	6	-8	114	5.18	*

Note: * the cluster with the peak in the pole STG showed a trend towards significance (p(FWE) < 0.06)

**Table 5:** Maternal brain activation in response to infant laugh sounds (LS) contrasted to control noise sounds (NCS). Cluster peaks of cerebral activity with brodmann areas (BA), coordinates in MNI space (*X*,* Y*,* Z*), cluster sizes, and *t*-values at *p*(FWE) < 0.05

Regions			MNI coordinates			Cluster size	*t*-value
			(peak within a cluster)				
	BA	Side	X	Y	Z		
Declive (fusiform)	37	L	-32	-66	-22	243	6.97
Putamen (hippocampus, amygdala)	38	R	30	-14	-8	523	5.91
STG sulcus (MTG)	22/21/41	R	52	-40	6	874	5.76
Putamen		L	-30	4	-8	132	5.36
Hippocampus (parahippocampus, midbrain SN)	34	L	-24	-14	-14	150	5.03
STG (Heschl’s gyrus)	22	L	-64	-22	10	261	4.78

**Table 6:** Maternal brain activation and deactivation in response to infant cry sounds (CS) contrasted to infant laugh sounds (LS). Cluster peaks of cerebral activity with brodmann areas (BA), coordinates in MNI space (*X*,* Y*,* Z*), cluster sizes, and *t*-values at *p*(FWE) < 0.05

	Regions		MNI coordinates				Cluster size	t-value
			(peak within a cluster)					
		BA	Side	X	Y	Z		
*Activated clusters*								
	Angular gyrus (supramarginal)	39/40	L	-48	-56	36	131	5.51
	MOG (Cuneus)	19/18	L	-24	-90	16	241	5.47
	Cerebellum Crus 1 (Declive)		R	26	-84	-28	131	5.33
*Deactivated cluster*								
	STG (MTG)	41	R	54	-18	2	119	-4.60

### Exploratory correlational analysis between brain reactivity to salient infant sounds and emotional availability (EA) scale and the caregiving involvement

Table 7 reports exploratory Spearman correlations between betas values from significant SPM contrasts and interest maternal EA, and caregiving involvement. As no correlations survived to the Bonferroni correction for multiple tests, the results should be considered with caution.

**Table 7:** Exploratory correlational analysis with beta values extracted from activated clusters in all contrast and maternal EA scales and caregiving involvement

		Maternal Emotional Availabilty								Caregiving Involvement	
		Sensitivity		Structuring		Non-intrusiveness		Non-hostility			
*Contrast [Infant Cry > Control Noise]*											
	STG (temporal pole)	0.09		0.44		0.20		0.27		0.03	
	Midbrain (bilateral hippocampus and amygdala)	0.12		0.49	*	0.12		0.23		0.11	
	pole STG (insula)	0.34		0.32		0.42		0.50	*	0.18	
*Contrast [Infant Laugh >Control Noise]*											
	Declive (fusiform)	0.51	*	0.34		0.58	**	0.45	*	0.37	
	Putamen (hippocampus, amygdala)	0.47	*	0.56	*	0.45	*	0.44	*	0.27	
	STG sulcus (MTG)	0.30		0.35		0.43		0.42		0.30	
	Putamen	0.43		0.54	*	0.55	*	0.14		0.46	*
	Hippocampus (parahippocampus, midbrain SN)	-0.02		0.26		0.10		0.12		0.30	
	STG (Heschl’s gyrus)	0.25		0.27		0.41		0.41		0.28	
*Contrast [Infant Cry > Infant Laugh]*											
	Angular gyrus (Supramarginal)	-0.31		-0.22		-0.29		-0.25		0.10	
	MOG (Cuneus)	-0.33		-0.14		-0.33		-0.07		-0.14	
	Crus 1 (Declive)	-0.59	**	-0.61	**	-0.51	*	-0.51	*	-0.20	
*Contrast [Infant Cry < Infant Laugh]*											
	STG (MTG)	0.07		0.21		0.25		0.26		0.21	

Note. * *p* < .05, ** *p* < .01, *** *p* < .001, two-tailed

## Discussion

The present preliminary study aimed to broaden current knowledge about the neural networks of parental caregiving in a sample of same-sex mothers. Indeed, although the traditional family structure no longer reflects the composition of families in contemporary society [56, 57, 58, 59, 60], there is a lack of research on the neurobiological basis of parenting in same-sex families, and data from same-sex mothers are particularly scarce. In accordance with established experimental protocols within the field (i.e [14, 50, 55, 62, 80, 81]), the present study was designed to assess the maternal brain response to significant infant cues, such as infant crying and laughing sounds, and its correlation with maternal involvement in childcare and maternal sensitivity during interaction with the child, operationalised here as emotional availability (EA) [46].

The results showed that the level of maternal involvement in childcare was correlated with the quality of mother-child interaction in mothers of same-sex families. Indeed, we found that mothers who were more involved in childcare had higher levels of sensitivity and non-intrusiveness in dyadic ecological interactions. We also found that children showed higher levels of responsiveness to the interactive contributions of the more involved mothers. The involvement in childcare, here conceived as the level of self-reported responsibility in different health related, nurturing and playful behaviours toward the child, contributes to the quality of the dyadic interaction, promoting affectivity, ability to understand and respond appropriately to the child’s signals, and ability to support the child’s activity without being too directive and limiting the child. These preliminary findings indicated a need for greater attention to be paid to the assessment of the extent to which parents are effectively involved in caring for their child in research on parenting quality.

From the brain analysis, we found that the infant cry, when compared to the control sound, preferentially activated brain regions with peaks in the right STG and right temporal pole extending to the insula, and a large brain region with a peak in the right midbrain extending bilaterally to the hippocampus and amygdala. Brain activations are consistent with previous research on parental brain and are related to auditory (STG), emotion regulation, motivation and salience processing [27, 38, 44, 53, 82]. Instead, we found that the infant’s laughter, when compared to the control sound, enhanced neural signal from brain areas with peaks in the right putamen, extending to the hippocampus and amygdala, and in the STS extending to the MTG. We also found increased brain activation in left-brain areas with peaks in the putamen, hippocampus extending to the parahippocampus and SN, and STG. Again, this pattern of brain activity is consistent with activity in regions involved in auditory elaboration, emotion processing and regulation, and parental motivation implicated in the parental brain model [83]. Although the neuroscientific evidence on brain activity associated with positive infant vocalisations is less systematic than that focused on infant crying, research has demonstrated that listening to infant laughter modulates activity in the amygdala, insula and temporal cortex [51, 52, 53, 84]. A cross-cultural study, investigating the common neural underpinnings of infant crying, reported that unfamiliar infant laughter, in contrast to a control noise stimulus similar to the one used in the present study, significantly enhanced maternal cerebral activation in the medial and lateral premotor frontal lobes, dopaminergic regions including the lentiform nucleus and putamen, insula and cerebellum [25]. Overall, our results so far are consistent with research in this area and add additional findings related to a different type of family structure, i.e. same-sex mothers. Although the findings on neural activation elicited by infant laughter appear to be consistent with theories of parental brain models, they should be treated with caution because the control sounds did not match the temporal pattern of each infant sound used in this study. Finally, we found that the infant’s cry, when compared to the infant’s laugher, enhanced neural signal from brain areas with peaks in the left angular gyrus with the supramarginal gyrus, the medium occipital gyrus extended to the cuneus, and in the right the cerebellum. Instead, the infant’s laugh activated more the primary auditory cortex (STG) extended to the MTG than the infant’s cry sound. Previous literature investigating differential brain activation patterns to unfamiliar infant vocalisations between parents and nonparents, suggested that parental whole-brain response was higher to infant crying than to infant laughter; in addiction, the STG was found to be affected by infant vocalisation in both parents and nonparents [53]. Further evidence in nonparent adults, showed that the temporal cortex was more activated by unfamiliar adult laughter than by crying [52]. In addition, administered level of oxytocin increased the connectivity between the amygdala and several regions underlying salience processing and emotion regulation, including the supramarginal gyrus, the precuneus together with the OFC, ACC and hippocampus [50]. The paucity of evidence for differential maternal brain responses to infant crying and infant laughter of familiar and unfamiliar children highlighted the need for further research.

More importantly, in this exploratory study, we expected that maternal involvement would exert an influence on the brain reactivity to infant vocalisations, based on the maturation of the experience of processing, interpreting and responding to one’s own child’s signals on a daily basis [85, 86, 87]. Indeed, we found a significant relationship between the degree of maternal involvement in childcare and the activation of the putamen in response to infant laughter sounds. Although maternal putamen activation in response to infant laughter has been previously reported in a cross-cultural study [25], no studies have assessed associations between brain response to infant laughter and maternal dimensions related to the involvement in daily caregiving behaviours. The putamen is a basal ganglia brain structure that has been consistently found to be involved in response to infant cry and that may play a role in the initiation of motivated behaviour and parental ritual behaviours [20, 44, 88]. If looking at the brain response to infant crying may be appropriate to assess the maternal adaptive response to infant needs during the early months postpartum [5, 31], the brain responses to positive vocalisations may be relevant to target in relation to other dimensions of parental care not strictly confined to child protection and caretaking. Laughter may be expected to elicit motivated behaviours aimed at maintaining dyadic closeness while providing support during daily activities in which the child is expected to participate. Consistent with this line of thinking, our results appear to show, for the first time, that the brain reactivity underlying the processing of positive vocalisations correlates with maternal involvement in pragmatic caregiving behaviours. These behaviours are characterised by the caregiver’s presence, which is related to both the physical and social care of the child.

We also found that the quality of EA during mother-child interaction demonstrated a systematic correlation with neural activity in response to both infant sounds, thus corroborating our initial expectations. Regarding brain response to infant crying, mothers who showed high levels of structuring and non-hostility during dyadic interaction showed increased activation in the right to left midbrain, extending to the bilateral amygdala, and in the right temporal pole to the insular cortex. The midbrain dopamine neurons are integral to the regulation of voluntary movement and the modulation of emotion-related behaviour, which is of paramount importance in the context of motivation to parenting [27, 89]. The temporal pole, together with the insula, is part of the limbic system, which is involved in processing emotional regulation and integrating emotional information from different modalities [90, 91, 92, 93]. According to a recent systematic review [94], a few neuroimaging studies have examined relationships between the construct of emotional availability and the brain response to infant emotional cues in normative parent samples. For example, EA dimensions of sensitivity, non-intrusiveness and non-hostility were found to be negatively correlated with the amygdala activation gradient in response to negative emotional infant stimuli (infant crying sounds and faces) [95, 96]. Further evidence from EEG data suggested instead that frontal brain activity related to inhibition response and cognitive tasks correlates positively with dimensions of structuring [97]. Optimal levels of EA structuring and non-hostility are dimensions that imply attuned and appropriate responses even to negative expressions of affection of the child [77, 98]. Referring to our results, we showed a significant preliminary correlation between the EA structuring scale and brain structures underlying parental motivation for the first time.

Exploratory correlations revealed a significant relationship between almost all maternal EA scales and activation of the bilateral putamen in the contrast between infant laughter and control noise. In addition, a significant correlation was found between activation of the right cerebellum in the infant cry/infant laugh contrast. Interestingly, the putamen activation was the only brain structure that correlated with the degree of caregiver involvement in childcare in our study. The putamen is a structure of the basal ganglia in the forebrain and is involved in a variety of functions ranging from movement control to learning, and is also involved in the elaboration of affective-cognitive processes that influence action and social behaviours [99, 100, 101, 102]. Indeed, the putamen, in conjunction with other structures of the basal ganglia, is involved in the processing of emotional vocalisations, exhibiting an increase in activation in response to happy vocalisations. Given its functional connection with the cerebellum [103], it contributes to the encoding of emotional stimuli for motivated actions [44]. Moreover, in the context of parent-child relationships, the putamen highly responds to the own child [104, 105] and, preferentially, to the happy faces [54]. In consideration of the aforementioned evidence, our findings indicate that mothers who are more engaged in childcare activities tend to demonstrate heightened EA. Furthermore, both high levels of EA and of childcare involvement are associated with greater activation of the putamen in response to positive vocalisations. This reflects a greater parental motivation to plan emotionally based and socially oriented behaviours.

In conclusion, our results would extend the current knowledge about the neurobiological bases of parenting by describing for the first time the brain responses of same-sex mothers to infant emotional vocalisations. On the other hand, data show that part of the evidenced brain responses to infant cues are positively associated with the quality of maternal behaviour during mother-child interaction, possibly confirming the connection between circuits of the so-called parental brain and parenting behaviour in ecological contexts. Moreover, the level of maternal involvement in childcare was associated to the specific brain reactivity to infant vocalisation as well as to the quality of maternal behaviour. It might then be that the daily shared mother-child social-affective experience modulates the functioning of the parental brain structures resulting in enhanced quality of dyadic relationship.

### Limitations and future research

The present study has a number of limitations. In particular, the sample size was insufficient to implement statistical predictive models to test how the level of involvement in childcare influences or mediates the relationship between the brain response to infant emotional cues and the maternal EA. In addition, given the small sample size, correlations were not corrected for multiple testing, so results should be considered exploratory and with caution. Indeed, we found that EA scales were moderately correlated with the brain response to negative and positive emotional vocalisations. The construct of sensitivity is derived from attachment theory and reflects certain aspects of parental response that directly influence the manner in which parents respond to infant cues [45, 46]. This, in turn, affects the brain’s reactivity to such cues [49, 54, 106]. In contrast, we found that the degree of involvement construct correlated more closely with the interactive quality of the caregiver, as assessed by the EA Scales [76]. It would be beneficial for future research to investigate how the degree of involvement in childcare may potentially moderate the quality of caregiver interactive skills in its significant relationship with adult brain reactivity to child cues [70, 107], moving beyond the parental role defined by the biological sex of the caregiver [63, 108].

Our study did not include a direct assessment of the maternal caregiver role, whether as the primary or secondary caregiver. Instead, a dimensional score was calculated to determine the percentage of time spent on specific parental activities [76]. Although a quantitative measure of the amount of time spent on different types of caregiving behaviours has been employed, it would be beneficial to assess additional dimensions that facilitate a more comprehensive evaluation of the caregiver’s role in supporting the child’s emotional development. To illustrate, at an implicit affective level, it would be beneficial to ascertain which caregiver is the preferred source of support for children in moments of high distress. The co-regulation of emotions and behaviour within the dyadic intersubjective space is fundamental to the child’s organisation of body-mind and affective experience, and ultimately to the child’s adaptive functioning and well-being [109].

Furthermore, the wide age range of the children constitutes a limitation of this study, as it encompasses various developmental stages. The rationale for using standardised infant vocalisations, previously assessed by an independent sample of both parents and nonparents, rather than, for example, using pictures of one’s own child for comparison with unfamiliar children, was partly to limit the risk of excessive diversity between child stimuli. In addition, the decision to exclude families with children under the age of three was made to overlook the hormonal effects typically associated with childbirth and breastfeeding in the early years of a child’s life [14, 20]. The above methodological choices were made with the idea of highlighting the potential link between intrinsic human sensitivity to biologically salient infant vocalisations and the quality of dyadic emotional availability with caregiving dimensions that reflect the mature and learned experience of parenting by the mothers in the present study [42, 62]. Future research would benefit from a larger sample size with a more stringent age range and longitudinal assessment to determine the direction and magnitude of the associations identified in this study. A larger sample would also facilitate the application of dyadic statistical models to account for the interdependency shared by mothers in couples. Furthermore, it is essential to consider that responses regarding childcare activities provided by same-sex mothers may be influenced by structural barriers, such as laws and policies, that vary by country. For instance, in the Italian context, parents who are not recognised as legal guardians of the child cannot perform certain activities alone with their child. Consequently, our study focused only on parental behaviours that could be carried out by both recognised and unrecognised mothers. In this regard, future studies should consider assessing sociocultural dimensions that can represent significant barriers for same-sex mothers, such as legal recognition and perceived social stigma.

Another limitation of the present study concerns the parity effect. The experimental protocol implemented did not allow to take into account the parity effect and no qualitative or quantitative data collection was carried out to capture the complexity of the caregiver’s experience in raising multiple children. We considered these variables as demographic information to correlate a posteriori with caregiving involvement, EA scale and brain response (Tables S1, S2 and S3). Therefore, our results cannot take into account the experience of multiparous mothers in terms of intrapsychic and behavioural aspects that contribute to determine the caregiving response, starting from the brain response to infant indices to caregiving behaviours and, in general, the way of being in relation with the child and the other caregiver [110, 111, 112].

Finally, another critical point was that in a very late postnatal period (as in our study, 3–11 years postpartum), mothers are more accustomed to responding to more mature child vocalisations to communicate social and needs requests to parents. Indeed, both crying and laughter from 3- to 11-year-old children are very likely to be associated with both verbalisations and behaviour/actions. In our study, both positive and negative vocalisations (infant sound stimuli) came from young children. However, if age had an effect, it should have affected the brain response to both types of infant sound stimulus in the same way. We did not expect the effect of age to be different for the sound of infant crying and infant laughter per se. However, if we consider the psychological measures used in our study (the scales of caregiver involvement in childcare and emotional availability), which refer more to the sharing of activities between mothers and children, it is possible that laughter, as a positive social cue that promotes emotional bonding, may correlate better with these measures.”

## Supplementary Information

Below is the link to the electronic supplementary material.

Supplementary Material 1

## Data Availability

Upon reasonable request, data and materials supporting the results or analyses presented in this study will be made available.
