# Supplementary material for: An exploratory fMRI study on the association of parental involvement in childcare with brain responses to infant vocalisations and dyadic interaction quality in same-sex mother families
Source: BMC Psychol. 2025 Oct 7;13:1116. doi: 10.1186/s40359-025-03439-9 (PMC12506274; doi:10.1186/s40359-025-03439-9)
Supplement: Supplementary file 1 — Supplementary Material 1 [file 40359_2025_3439_MOESM1_ESM.docx]

**Table S1.** Correlational analysis between Demographics and Caregiver Involvement

|  | **1** | **2** | **3** | **4** | **5** | **6** | **7** | **8** |  |
| --- | --- | --- | --- | --- | --- | --- | --- | --- | --- |
| **1. Age mother** | – |  |  |  |  |  |  |  |  |
| **2. Age child** | .56** | – |  |  |  |  |  |  |  |
| **3. Child sex** | -.28 | -.09 | – |  |  |  |  |  |  |
| **4. Parity (number of children)** | .13 | .23 | -.09 | – |  |  |  |  |  |
| **5. Educational level** | -.05 | -.21 | .13 | .18 | – |  |  |  |  |
| **6. Relationship duration (partner)** | .15 | .65** | .18 | .13 | -.13 | – |  |  |  |
| **7. Biological parent** | .11 | .12 | -.06 | -.03 | .02 | .18 | – |  |  |
| **8. Caregiver Involvement** | .01 | .22 | .14 | -.08 | -.44* | .21 | .23 | – |  |

*Note1*. * p < .05, ** p < .01, *** p < .001

*Note2.* The variable biological mother referred only to mothers who gave birth

**Table S2.** Correlational analysis between Demographics and Emotional Availability (EA) Scale

|  | **1** | **2** | **3** | **4** | **5** | **6** | **7** | **8** | **9** | **10** | **11** | **12** | **13** |  |
| --- | --- | --- | --- | --- | --- | --- | --- | --- | --- | --- | --- | --- | --- | --- |
| **1. Age mother** | – |  |  |  |  |  |  |  |  |  |  |  |  |  |
| **2. Age child** | .56** | – |  |  |  |  |  |  |  |  |  |  |  |  |
| **3. Child sex** | -.28 | -.09 | – |  |  |  |  |  |  |  |  |  |  |  |
| **4. Parity (number of children)** | .13 | .23 | -.09 | – |  |  |  |  |  |  |  |  |  |  |
| **5. Educational level** | -.05 | -.21 | .13 | .18 | – |  |  |  |  |  |  |  |  |  |
| **6. Relationship length (partner)** | .15 | .65** | .18 | .13 | -.13 | – |  |  |  |  |  |  |  |  |
| **7. Biological parent** | .11 | .12 | -.06 | -.03 | .02 | .18 | – |  |  |  |  |  |  |  |
| **8. Sensitivity** | -.30 | .02 | -.15 | -.27 | -.13 | .14 | .12 | – |  |  |  |  |  |  |
| **9. Structuring** | -.23 | -.12 | -.20 | -.36 | -.04 | .19 | .20 | .79*** | – |  |  |  |  |  |
| **10. Nonintrusiveness** | -.34 | .17 | -.02 | -.18 | -.37 | .45* | .22 | .80*** | .69*** | – |  |  |  |  |
| **11. Nonhostility** | -.09 | .10 | .04 | -.20 | .15 | .28 | .04 | .70*** | .53* | .48* | – |  |  |  |
| **12. Responsiveness** | -.35 | -.07 | -.28 | -.28 | -.17 | -.03 | -.06 | .81*** | .74*** | .70*** | .46* | – |  |  |
| **13. Involvment** | -.32 | -.19 | -.26 | -.47* | -.22 | -.070 | -.03 | .77*** | .80*** | .64** | .47* | .91*** | – |  |

*Note1*. * p < .05, ** p < .01, *** p < .001

*Note2.* The variable biological mother referred only to mothers who gave birth

**Table S3.** Correlational analysis between parity variable (number of children, n_max_ = 2) and beta values extracted from activated clusters in all contrast.

|  |  |  |  |
| --- | --- | --- | --- |
|  |  | **Parity** | **p** |
| *Contrast [Infant Cry > Control Noise]* | |  |  |
|  | STG (temporal pole) | -0.10 | 0.652 |
|  | Midbrain (bilateral hippocampus and amygdala) | -0.37 | 0.103 |
|  | pole STG (insula) | 0.14 | 0.547 |
|  |  |  |  |
| *Contrast [Infant Laugh > Control Noise]* | |  |  |
|  | Declive (fusiform) | 0.04 | 0.881 |
|  | Putamen (hippocampus, amygdala) | -0.19 | 0.406 |
|  | STG sulcus (MTG) | 0.17 | 0.45 |
|  | Putamen | -0.26 | 0.253 |
|  | Hippocampus (parahippocampus, midbrain SN) | -0.24 | 0.287 |
| *Contrast [Infant Cry > Infant Laugh]* | |  |  |
|  | Angular gyrus | -0.10 | 0.764 |
|  | MOG | 0.21 | 0.363 |
|  | Cerebellum crus1 | 0.44 | 0.049 |
| *Contrast [Infant Cry < Infant Laugh]* | |  |  |
|  | STG | 0.23 | 0.324 |
|  |  |  |  |

*Note*. * p < .05, ** p < .01, *** p < .001
